# Medical Cannabis and Industrial Hemp Tissue Culture: Present Status and Future Potential

**DOI:** 10.3389/fpls.2021.627240

**Published:** 2021-03-03

**Authors:** Dinesh Adhikary, Manoj Kulkarni, Aliaa El-Mezawy, Saied Mobini, Mohamed Elhiti, Rale Gjuric, Anamika Ray, Patricia Polowick, Jan J. Slaski, Maxwell P. Jones, Pankaj Bhowmik

**Affiliations:** ^1^Department of Agricultural, Food, & Nutritional Sciences, University of Alberta, Edmonton, AB, Canada; ^2^Canadian Cannabis Breeding Consortium, Edmonton, AB, Canada; ^3^InnoTech Alberta, Vegreville, AB, Canada; ^4^Farmers Business Network Inc., Winnipeg, MB, Canada; ^5^National Research Council, Saskatoon, SK, Canada; ^6^Department of Plant Agriculture, University of Guelph, Guelph, ON, Canada

**Keywords:** *Cannabis sativa*, micropropagation, tissue culture, hemp, *in vitro*

## Abstract

In recent years high-THC (psychoactive) and low-THC (industrial hemp) type cannabis (*Cannabis sativa* L.) have gained immense attention in medical, food, and a plethora of other consumer product markets. Among the planting materials used for cultivation, tissue culture clones provide various advantages such as economies of scale, production of disease-free and true-to-type plants for reducing the risk of GMP-EuGMP level medical cannabis production, as well as the development and application of various technologies for genetic improvement. Various tissue culture methods have the potential application with cannabis for research, breeding, and novel trait development, as well as commercial mass propagation. Although tissue culture techniques for plant regeneration and micropropagation have been reported for different cannabis genotypes and explant sources, there are significant variations in the response of cultures and the morphogenic pathway. Methods for many high-yielding elite strains are still rudimentary, and protocols are not established. With a recent focus on sequencing and genomics in cannabis, genetic transformation systems are applied to medical cannabis and hemp for functional gene annotation via traditional and transient transformation methods to create novel phenotypes by gene expression modulation and to validate gene function. This review presents the current status of research focusing on different aspects of tissue culture, including micropropagation, transformation, and the regeneration of medicinal cannabis and industrial hemp transformants. Potential future tissue culture research strategies helping elite cannabis breeding and propagation are also presented.

## Introduction

Cannabis is a multipurpose crop with nutritional, medicinal, and industrial uses. Its leaves and flowers produce a spectrum of biologically active secondary metabolites, seeds are a source of nutritious oil and protein, and the stem contains two types of fiber serving as feedstock for the manufacturing of a variety of bio-based consumer goods (Small, 2004; Rodriguez-Leyva and Pierce, 2010; Wargent et al., 2013; Andre et al., 2016; Musio et al., 2018). The crop may have originated and been domesticated over 5000 years ago in Asia; since then, it has been interwoven with human history. In the South Asian regions, cannabis biotypes with elevated THC levels were commonly used for medicinal and recreational purposes, building a strong connection to social and religious rituals. While in the temperate climates, low-THC types were grown initially for fiber, and later also for food (Cheng, 1963; Li, 1974; Mechoulam, 1986; Cherney and Small, 2016; Clarke and Merlin, 2016; Jiang et al., 2016). Since the discovery of two cannabinoids [cannabidiol (CBD) in 1963) and tetrahydrocannabinol (THC) in 1964] in Dr. Raphael Mechoulam’s laboratory, more than 100 additional phytocannabinoids, flavonoids, and over 150 terpenes have been identified in the plant (Andre et al., 2016; Booth and Bohlmann, 2019; Rea et al., 2019). This high-value crop has built a strong foundation for a multi-billion-dollar global industry. Due to legal restrictions, research and development work has been slow and prevented researchers from investigating cannabis openly and making use of its full potential.

Recent cannabis legalization amendments in Canada, Europe, some parts of the United States, and other parts of the globe have helped promote research and use of this multipurpose crop. Commercial production increased in anticipation and response to the federal legalization of cannabis in Canada in October 2018 under the Cannabis Act (Government of Canada, 2018). Canada became the second nation after Uruguay (legalized December 2013) to legalize cannabis for recreational use at the federal level (Adinoff and Reiman, 2019). In the United States, 12 states have legalized cannabis for recreational use, with another 22 legalizing medical use (Adinoff and Reiman, 2019).

Inherently, cannabis is a dioecious species, with male and female flowers found on separate plants. Monoecious forms, which produce male and female flowers on the same plant, are very seldomly found in nature (Clarke and Merlin, 2016). Commercial monoecious cultivars of hemp have been bred for oilseed production and improved fiber yield and uniformity that cannot be achieved in dioecious forms exhibiting asynchronous maturation of the stems, as male plants commence an accelerated aging process soon after pollen shed. Due to the dioecious nature of most high THC-type cannabis and the lack of advanced breeding to produce true-to-type seed, they are propagated vegetatively and often grown indoors. Vegetative propagation maintains genetic purity and uniformity among the plants. Traditionally, indoor cannabis cultivators have depended on cuttings from a mother plant to produce genetically similar plants. While cannabis generally roots well (Caplan et al., 2018) and stem cuttings can produce large numbers of genetically similar plants, this method requires significant amounts of space. It has been observed that plants become less vigorous over time, the mother plants are susceptible to pests and diseases, and the resulting cuttings can harbor unwanted disease and serve as primary inoculum in production spaces.

As an alternative, *in vitro* techniques offer a promising approach for mass production and germplasm maintenance (Withers and Engelmann, 1997; Watt et al., 2000). Micropropagation can facilitate high throughput propagation in many species and forms the basis of disease-free plants for certified clean plant programs (Lineberger, 1983; Al-Taleb et al., 2011). Tissue culture based clean plant programs have been used in other vegetatively propagated crops such as potatoes, sweet potato, dates, sugarcane, banana, rice, tobacco, strawberry, grapes, orchids, roses, fruit trees, and some more horticulture of food and ornamental crops, helping to eradicate or prevent the spread of many plant pests, diseases, and viruses (National Clean Plant Network, 2020). Thus, developing an optimized *in vitro* method for propagating clean plants is a crucial strategy to produce large-scale genetically identical plants, retain genetic integrity, and maintain the long-term sustainability of the economically valuable crop (Conway, 2012). This review article aims to provide a comprehensive overview of the most updated available scientific research reported to date on tissue culture in cannabis, to contribute to our understanding of the cannabis tissue culture, and to assess potential applications of the optimized techniques in cannabis plant propagation, regeneration, and transformation.

## Industrial Hemp vs. Medical Cannabis (Marijuana)

According to Small et al. (1976), there are four groups of cannabis, ‘non-intoxicant (some *C. sativa* accessions),’ ‘semi-intoxicant’ (some *C. sativa* accessions), ‘intoxicant (*C. indica*),’ and ‘wild’ (*C. ruderalis*). *Cannabis* includes *C. indica, C. ruderalis*, and *C. sativa*. However, it has also been proposed that these three groups all belong to a single species (*C. sativa*) and the taxonomic classification among these proposed species remains a debated issue in *Cannabis* taxonomy (McPartland, 2018). For morphological and chemical characters (i.e., floral morphology and THC content), the earlier report considered them as different subspecies (Small and Cronquist, 1976), while another classified them as different species (Hillig, 2005).

Further complicating matters is the legal distinction between hemp and drug (narcotic) type cannabis. Any plant containing less than a defined concentration of the psychoactive THC is classified as hemp. In contrast, anything above the critical limit is classified as drug type cannabis. Depending upon the jurisdiction, the threshold THC concatenations in flowering plant parts differentiating between industrial hemp and drug type cannabis range from 0.2% of dry weight in most European counties, which is 0.3% in Canada, United States, and China and Brazil to 1% in Switzerland, Uruguay, Columbia, Mexico, and several Australian states. While this distinction is not based on taxonomy or genetic relationships, several studies have shown that most hemp cultivars are genetically distinct from drug-type cannabis (Rotherham and Harbison, 2011; Cascini et al., 2019). Mainly due to legal restrictions, artificial selection influenced by a decade’s long black market, and insufficient knowledge of the *Cannabis* taxonomy, these sub-types are poorly defined, especially the drug type cannabis.

Hemp is generally cultivated from seed and has named cultivars similar to most other crops. In contrast, drug type cannabis is generally propagated clonally; the clones are often referred to as ‘strains’ but are also often referred to as cultivars. As such, any given strain/cultivar can produce various clonal accessions with dramatically different phenotypes, making names unreliable. Further, many strains are offered by different seed companies, and the degree of genetic similarity or difference among providers has not been quantified; therefore, it is generally expected and accepted that there is significant variation within a single strain among seed companies and even within seed lots. Due to these factors, strain names in drug type cannabis are not reliable regarding a uniform phenotype.

**FIGURE 1:**
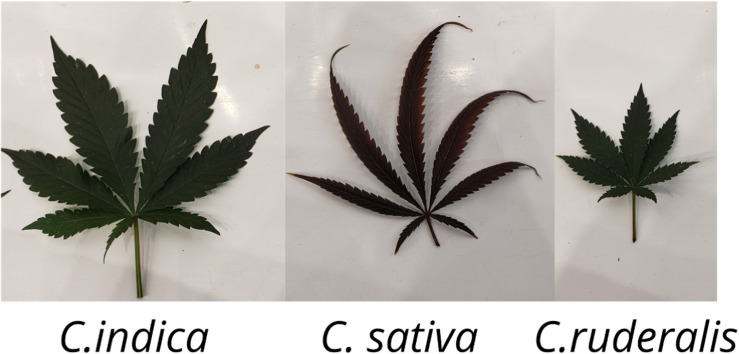
Cannabis leaf showing morphological differences of the three different species (*C. indica, C. sativa*, and *C. ruderalis*).

*Cannabis indica* and *Cannabis sativa* are the major sources of cannabinoids, and are predominantly cultivated, while the third species, *C. ruderalis* is a wild and hardy species and is rarely grown by cultivators as there is no significant content of cannabinoids (Hilling and Mahlberg, 2004). In many lay literatures, distinction of ‘indica’ and ‘sativa’ have been mentioned and some of the earlier publications have also gathered some phenotypic differences (Table 1 and Figure 1); however, there is neither solid taxonomic agreement nor genetic or chemical evidence supporting the differences (Gloss, 2015; Sawler et al., 2015; Piomelli and Russo, 2016). The use of ‘indica’ and ‘sativa’ is vaguely based on the general notion that ‘sativa’ originated from European hemp, while ‘indica’ originated from the Indian subcontinent (Small, 2015), but their exact origin is still debatable.

**TABLE 1:** Phenotypic differences among *C. indica, C. sativa*, and *C. ruderalis* ecotypes.

**Trait**	***C. indica***	***C. sativa***	***C. ruderalis***
Climate	Tropical intense sunlight, cool arid regions (Afghanistan, Pakistan, Northern India, Nepal)	Subtropical humid climate, more rainfall, In Mexico, Colombia, Nigeria, Thailand	Northern climates, cool and hilly places, grows in wild (Russia, China)
Height	Short 1– 2 m	Tall up to 3–4 m	Very short bushy 0.6–1.0 m
Cannabis female flower	Compact and short inflorescence	Loose packed and long inflorescence	Small, compact, very short inflorescence
Habit	Shorter internode	Longer internode	Very short internodes
Leaves	Broad	Narrow	Smaller leaves
Leaf color	Dark green	Light green	Dark leaves
Stalk	Shorter woody	Taller, fibrous	Short fibrous
Maturity	Early maturity 2–3 months	Late maturity 4–6 months	Very early maturity 1.5–2 months, autoflowering
Root system	Condensed root system	Deep, expansive	Shallow smaller
Cannabinoid content	Lower THC, could be higher CBD	High THC, Lower CBD in general	Low THC and CBD
Effect	Relaxing effect, inflammation reduction (Medical use preferred)	Incite euphoria, head high (stress relief, recreational use preferred)	Not grown commercially, only for breeding earliness

*Information derived from Schultes et al. (1974); Small and Cronquist (1976), Hillig (2004); Clarke and Merlin (2013), Farag and Kayser (2017), and Small (2017).*

## Traditional Cloning in Cannabis

**FIGURE 2:**
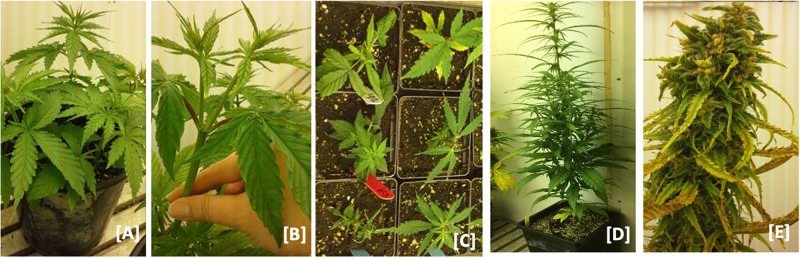
Hemp nodal cloning. **(A)** Hemp plants at 6–8 leaf stage. **(B)** Elongated lateral branches after terminal buds removed from female plants **(C)** lateral branches planted in soil after excision from mother plants and. **(D)** Vegetative clones transferred to 7-inch pots after roots were established and grown. **(E)** Vegetative clone at maturity.

For decades, seed propagation in cannabis has supported agricultural needs and facilitated genetic improvement. However, with modern horticultural practices to the cannabis industry, stem cutting or traditional cloning, and *in vitro* propagation of this high-value crop has become a common practice (Lata et al., 2009a,b, 2011; Potter, 2009). Other methods of propagation are encapsulation of axillary nodes in calcium alginate beads (Lata et al., 2009a), leaf derived callus (Lata et al., 2010c), and temporary immersion bioreactor systems (Lata et al., 2010b) but these are limited in lab experiments only. Traditional cloning involves taking stem cuttings from a healthy mother plant and providing a rooting environment for the newly cut clone (Figure 2). For selection as a donor, a clear indication of alternating branches with no visible sign of insects, fungus, or any mineral deficiency in a mother plant is required. Cuttings can be taken from any part of a donor; despite some suggestions that growth in the lower half is better, no difference was observed between cuttings taken from the upper and lower part of the plant (Caplan et al., 2018). However, further research is warranted to test this across more genotypes and conditions. In general, cannabis propagates readily from stem cuttings even without rooting hormones.

Stem cuttings have advantages over seed propagation, including quicker maturation, true-to-type plants, and elite genetics maintenance (Table 2). Along with the ease of propagation, the practice can limit unwanted gene flow (McKey et al., 2010), for example, between the hemp and drug-type, potentially retaining the proportions of active metabolites.

**TABLE 2:** Comparison between tissue culture cloning, manual cloning, and seed propagation in cannabis.

**Propagation system**	**Seed**	**Traditional cloning**	***In vitro***
Roots	Tap root is prominent, grow deep, suitable for field cultivation	Adventitious roots grow from stem laterally, suitable for indoor cultivation	Adventitious roots grow from stem laterally, suitable for indoor cultivation
Genotype	In hybrids, genotype is different for each seed. In feminized seeds, genotype is close to each other	Same as mother plant	Same as mother plant
Rooting hormone	Not required	0.1% Indole-3-butyric acid (IBA) is used to promote rooting	0.1% IBA is used to promote rooting
Sexual type	Segregate in male and female (about 50% each in case of hybrid seeds); near 100% female in case of feminized seeds	All female but chances of developing hermaphrodites or mutated males	All female but chances of developing hermaphrodites or mutated males
Preferred growing	Outdoor	Indoor, hydroponic, aeroponic 18:6 h photoperiod	Indoor/hydroponic/aeroponic 18:6 h photoperiod
Preferred Light Condition	Variable between 500 – 2500 μmolm^2^ s^–1^	Variable between 200 and 300 μmolm^2^ s^–1^	Variable between 50 and 100 μmolm^2^ s^–1^
Yield	500–600 g/plant, relatively long growing cycle and high vegetative growth	40–60 g/plant, relatively small plant, short growing cycle, flower matures within 2 months	40–60 g/plant, relatively, small, short growing cycle, flower matures within 2 months
THC%	<0.3% THC; mostly used for propagating industrial hemp	Between 4 and 30% THC depending on strain	Between 4 and 30% THC depending on strain
Growing medium	Soil/compost	Compost/vermiculite cubes/rockwool/hydrotone clay balls	Sterilized tissue culture medium
Clone health	Chances of seedling infection with mites, sucking pests, powdery mildew, Hop latent viroid (Dudding disease)	Lower chances as grown under controlled condition but could carry disease or pests if cutting come from infected mother plants. If mother plant was infected or symptomless carrier for Hop latent viroid (Dudding disease), chances to carry it forward	Lowest chances as grown under clean condition to carry disease or pests as multiplied from clean stock. Opportunity to clean for Hop Latent virus as coming from nodal clone stocks free of Hop latent viroid (Dudding disease)
Storage	2–3 years in cool dry place	In a dome for a week	For a week in controlled condition and up to 12 months at 4°C
Storage requirements	Protective cover from high sunlight, temperature, and wind; watering as necessary	Cuttings require 65–75% relative humidity; 20–23°C temperature and artificial light for growth; proper ventilation	Controlled and clean purified air HEPA filtered air in culture rooms; 45–50% relative humidity in culture rooms; 20–22°C temperature, effective ventilation
Multiplication rate	One plant can yield thousands of seeds under open pollination/between 100–200 seeds from a feminized plant	150–200 clones from one month grown vegetative plant	One to four multiplication rates in one month period but grows exponential in number with time
Hardening requirement	Not necessary	About 2–3 days; cuttings are little easier to root and acclimatize in growing environment	About a week, transition from culture tubes to soil/compost is little riskier
Cost effectiveness	Can be grown outdoor under little care	Simpler indoor setup	Tissue culture lab investment
Preferred use	Field	Recreation cannabis	Medical Cannabis

*Information derived from Chandra et al. (2008, 2015), Caplan (2018), and Chandra et al. (2020).*

On the downside, space for large scale production is a concern as it can take considerable physical space, representing as much as 20–25% of production space just for cloning. Also, since it is currently manually performed, there is a low multiplication rate, and it is expensive in the long run. Therefore, this technique is more suitable for small growers requiring less than 1000 plants per growth cycle. For this reason, an adaptable, scalable, and robust high throughput tissue culture system with a high multiplication rate which preserves cannabis genetics, and produces more vigorous plants than manual clones, can prove to be more cost-effective in the long run (Table 2). Even small- scale growers with a small budget to use this technique to preserve genetics and test their desired strains’ regenerative capacity as a proof-of-concept. Building a team of experts to develop and execute tissue culture protocols successfully can be expensive and time-intensive initially; however, in the long term, it is a promising tool that has benefited many industries, including horticulture and cereal crops (Brown and Thorpe, 1995; Hussain et al., 2012).

Stem cuttings or traditional cloning method is the widely used propagation system adopted by many growers. *In vitro* propagation is establishing in cannabis industry slowly and is expected to take over the traditional cloning method. Although stem cuttings and *in vitro* clones can be comparable in terms of vegetative growth and physiological performance (Lata et al., 2009a), *in vitro* clones provide many advantages such as faster multiplication rate, clean clones without disease or virus, cost effective etc. (Table 1). Considering these advantages *in vitro* propagation is expected to become method of choice for propagation as well as genetic preservation in cannabis in the near future.

## Current Utilization and Opportunities for Cannabis Tissue Culture

The legal hemp for CBD production and the medical cannabis industry is a fast- growing market, and cultivators are turning toward advanced scientific approaches such as *in vitro* micropropagation, to reduce the production costs and offer scalable, healthy, and high-quality cannabis variety. In addition to a critical need for cost-effective propagation to meet demand, there is also a desire to establish and properly characterize cultivars equivalent to those of traditional agriculture with specific, consistent THC and cannabinoid content to match particular drug and therapeutic requirements. Legalization has opened up the options for accessing more mainstream research applications. This increases the demand for the application of some additional cell technologies applications to this crop.

### *In vitro* Micropropagation

**FIGURE 3:**
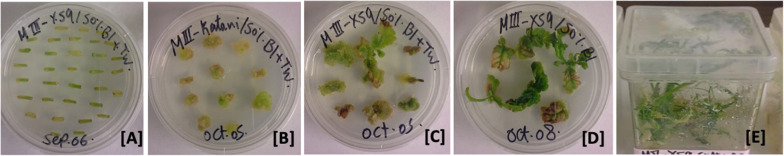
Hemp tissue culture propagation. **(A)** Hypocotyl explants on callus-induction media. **(B)** Hypocotyl explants with the callus on callus induction media. **(C,D)** Callus and developing shoots on shoot-induction media. **(E)** Developed shoots on root-induction media.

Although a few hemp cultivars have regenerated *in vitro* (Figure 3), *Cannabis* spp. have gained a wide reputation for being recalcitrant to tissue culture. At the beginning of the 1970s, along with the conventional propagation system, *in vitro* cultures of cannabis were initiated. The majority of the earlier *in vitro* studies were focused on cannabis callus culture to produce cannabinoids (Veliky and Genest, 1972; Itokawa et al., 1975, 1977; Hemphill et al., 1978; Hartsel et al., 1983; Loh et al., 1983; Fisse and Andres, 1985). Although there are multiple reports on shoot proliferation via micropropagation (Table 3), there are fewer scientific reports showing regeneration of a full plant through *de novo* regeneration (Mandolino and Ranalli, 1999; Slusarkiewicz-Jarzina et al., 2005; Wielgus et al., 2008; Chaohua et al., 2016).

**TABLE 3:** Cannabis cell culture, transformation, and micropropagation work since 1972–2020.

**Species**	**Cultivar**	**Study type**	**Explant**	**Organogenesis/Transformation**	**References**
*Cannabis sativa*	Unknown	Cell suspension culture for active metabolites	Seedling tissues	No/No	Veliky and Genest, 1972
*Cannabis sativa*	Unknown	Assessment of cannabinoids and essential oil in callus	Seedling tissues	No/No	Itokawa et al., 1975
*Cannabis sativa*	Unknown	Biotransformation of cannabinoid precursors using suspension cultures	Seedling tissues	No/No	Itokawa et al., 1977
*Cannabis sativa*	Drug type (‘152 Strain’); Fiber type (‘150 Strain; TUA(2):C-71)	Cannabinoid content in callus	Bracts, calyx, and leaf tissues	No/No	Hemphill et al., 1978
*Cannabis sativa*	Unknown	Root development from callus	Seedling	Yes/No	Fisse et al., 1981
*Cannabis sativa*	Unknown	Callus culture	Seedling tissue	No/No	Heitrich and Binder, 1982
*Cannabis sativa*	Unknown	Assessment of metabolites inducing callus and suspension culture	Embryo, leaf, and stem	No/No	Loh et al., 1983
*Cannabis sativa*	Unknown	Biotransformation of cannabinoid by cell suspension culture	Seedling tissues	No/No	Hartsel et al., 1983
*Cannabis sativa*	Unknown	Callus induction	Stem, cotyledon, and root	No/No	Fisse and Andres, 1985

**Species**	**Cultivars**	**Study type**	**Explants type**	**Organogenesis/Transformation**	**References**

*Cannabis sativa*	Hemp type	Rooting and shooting from clone cuttings	Axillary shoots	Yes/No	Richez-Dumanois et al., 1986
*Cannabis sativa*	Unknown but high THCV	Biotransformation of cannabinoids using cell culture method	Leaf tissues	No/No	Braemer and Paris, 1987
*Cannabis sativa*	Hemp type	Preservation procedure of cannabis suspension cultures	Floral part	No/No	Jekkel et al., 1989
*Cannabis sativ*	Hemp	Callus formation from all the test tissues;	Leaf, hypocotyl, cotyledon, and root	Yes/No	**Mandolino and Ranalli, 1999
*a*		shoot regeneration from hypocotyl, cotyledon, and root			
*Cannabis sativa*	Fedora 19, Felina 34	Regeneration of root from callus but no shoot.	Explant not identified	Yes/Yes (Information not descriptive)	Mackinnon et al., 2000
*Cannabis sativa*	Hemp type	Gene transformation and Callus formation	Stem and leaf	No/Yes	Feeney and Punja, 2003
*Cannabis sativa*	Silesia (m), Fibrimon-24 (Potential monoecious), Novosadska, Juso-15, Fedrina-74 (m)	Full plant regeneration from callus	Petiole, axillary bud callus, and callus from internodes	Yes/No	**Slusarkiewicz-Jarzina et al., 2005
*Cannabis sativa*	Beniko (m), Bialobrzeskie (m)	Regeneration of Hemp	Roots, leaves, and stems	Yes/No (only abstract is available in the public database)	Plawuszewski et al., 2005
*Cannabis sativa*	Bealobrzeskie (m), Beniko (m), Silesia (m)	Callus induction and plant regeneration	Stem and cotyledon	Yes/No	^∗∗^ Wielgus et al., 2008
*Cannabis sativa*	Hemp type	Regeneration of shoot from meristems	Cotyledon, stem, and root	Yes/No	Casano and Grassi, 2009
*Cannabis sativa*	Hemp type	Cell suspension culture for secondary metabolites	Leaf tissues	No/No	Flores-Sanchez et al., 2009
*Cannabis sativa*	MX-1	Direct organogenesis using nodal segments; synthetic seed development.	Nodal segments	Yes/No	Lata et al., 2009a
*Cannabis sativa*	Changtu	Shoot tip culture	Shoot tip	Yes/No	Wang et al., 2009
*Cannabis sativa*	MX	Regeneration from leaf derived callus	Leaf tissue	Yes/No	Lata et al., 2010c
*Cannabis sativa*	MX	Synthetic seeds for conservation of clones	Nodal segments	Yes/No	Lata et al., 2011
*Cannabis sativa*	Futura77, Delta-llosa, Delta405	*Agrobacterium* infection of cannabis roots	Hypocotyls, cotyledon and cotyledonary node	Yes/Yes	Wahby et al., 2013
*Cannabis sativa*	unidentified	Regeneration of plants from callus	Leaf	Yes/No	** Hussain, 2014 (Thesis)
*Cannabis sativa*	Long-ma No. 1	Micropropagation	Internodes	Yes/No	Jiang et al., 2015
*Cannabis sativa*	Unidentified	Callus induction and Shoot regeneration from callus	Cotyledon and epicotyledon	Yes/No	**Movahedi et al., 2015
*Cannabis sativa*	Unidentified	Cell culture	Root	No/No	Farag and Kayser, 2015
*Cannabis sativa*	Changsa	Full Plant regeneration from callus	Cotyledon	Yes/No	Chaohua et al., 2016
*Cannabis sativa*	Hemp type	Direct organogenesis: in vitro root and shoot proliferation	Nodal segments	Yes/No	Lata et al., 2016
*Cannabis sativa*	Bialobrzeskie and Monica	Direct organogenesis (shoot and roots) using phytohormones	Shoot tips	Yes/No	Grulichova et al., 2017
*Cannabis sativa*	Wappa	Direct organogenesis (rooting success of stem cuttings)	Stem cuttings	Yes/No	Caplan et al., 2018
*Cannabis sativa*	Unknown	Cannabis transformation and regeneration	Leaf segments (for micropropagation), protoplast (transformation), and pollen (transformation)	Unclear/Yes	Flaishman et al., 2019 (Patent)
*Cannabis sativa*	Hemp Landrace, Futura, Canda, Joey, CFX-2 and Cherry × Workhorse	Determination of optimal hormone and mineral salts for callus induction in hemp.	Stem cuttings	Yes/No	Thacker et al., 2018
*Cannabis sativa*	Medicinal cannabis but strain unknown	Assessment of cannabis shoot tips for their rooting efficiency	Shoot tips and nodal cuttings	Yes/No	Kodym and Leeb, 2019
*Cannabis sativa*	High THC accessions (1KG2TF, S1525, H5458)	Regeneration of shoots from immature and mature inflorescence	Floral tissues	Yes/No	Piunno et al., 2019
Cannabis sativa	Finola and Euphoria	Callus culture; direct regeneration, and gene transformation	Leaves, petiole, and auxiliary buds	No/Yes	Schachtsiek et al., 2019
Cannabis sativa	Tygra, Monoica, Bialobrzeskie, Fibrol	Direct organogenesis (roots) from cuttings	Seedlings	Yes/no	Smýkalová et al., 2019
Cannabis sativa	Unknown	Production of phytocannabinoids from cell culture	Leaf tissue	No/No	Whitton, 2019, 2020 (Patent)
Cannabis sativa	Drug type C. sativa (BA-1)	Media optimization for callogenesis and micropropagation using explants from both male and female strains	Leaf and nodal explants	Yes/No	Page et al., 2020 (preprint)
Cannabis sativa	Hemp, Ferimon (m), Felina32 (m), Fedora17 (m), USO31 (m), and Finola	*In vitro* plant regeneration and ploidy levels of regenerated plants	Cotyledon, hypocotyl	Yes/No	**Galán-Ávila et al., 2020

The letter ‘m’ indicates the monoecious cultivar. **Indicates studies with the regeneration of shoot or root or both from a single cell.

**FIGURE 4:**
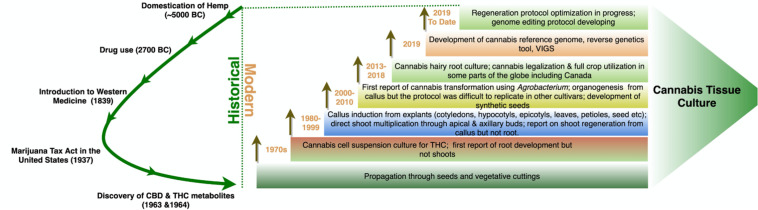
Evolution of cannabis tissue culture research. The green curved arrow on the left shows the key events in cannabis use. Each rectangle on the right shows the major research and development activities at different years. Each brown arrow indicates that the technology is continuously developing and research work is in progress in the particular research area.

The majority of regenerated strains and cultivars were monoecious, with few dioecious lines (Table 3). Recently, the optimization of a micropropagation and callogenesis protocol was reported for a few medical cannabis genotypes (Page et al., 2020). Although 48 years passed (Figure 4) since the first report of *in vitro* cell culture in cannabis, the available protocols are limited and inconsistent. *In vitro* regeneration of a cannabis plant from a single cell is still a challenge. Thus, the multi-billion-dollar cannabis industry needs an optimized tissue regeneration protocol for both industrial and medical cannabis.

It is generally understood that the most experienced cannabis companies have developed tissue culture and micropropagation techniques over the last two decades. However, most achievements in this *in vitro* field are held as a trade secret because of the competitive advantage provided within the industry. The most crucial challenges for the cannabis success micropropagation have been how to (i) reduce the length of subculture to minimize the occupied time and space, (ii) induce better root systems to increase the survival rate to >95%, (iii) optimize Plant Growth Regulators (PGRs), light (intensity and quality) and temperature required to maintain the genetically stable true-to-type clones. A generalized micropropagation workflow would require 7–8 weeks of culture transfer, 3 weeks of shoot multiplication, and 4 weeks of rooting. In terms of PGRs application, the best recommendation is optimized cytokinin and auxin for the vegetative medium and no cytokinin for the rooting medium using full MS media.

In recent years Canadian Licensed producers who are research-oriented have overcome some of these challenges. For example, the acclimatization period has been significantly reduced to less than 3 weeks. Another micropropagation challenge that the cannabis industry has recently solved is optimizing light intensity, light quality, and photoperiod in the culture room and maintaining the most effective temperature during shoot growth and root formation. Some unpublished data shows an increased propagation rate, from 3.5 to 5.8, during sub-culturing from each plantlet, through understanding and obtaining the right abiotic conditions within the culture room. As a starting point, some successful protocols are implemented with the minimum risk of somaclonal variation in cannabis (Movahedi et al., 2015; Lata et al., 2016, 2017; Grulichova et al., 2017; Page et al., 2020). These are game-changing procedures toward commercialization for cannabis micropropagation at a large-scale operation facility.

### Genetic Transformation

An ability to identify, characterize, and apply the genetic variability using biotechnology is the basis of molecular breeding. There are forward and reverse genetics approaches for genetic studies of an uncharacterized allele. With the improvement of sequencing technology, genetic transformation using reverse genetic tools has been an advantage in the molecular breeding program. While cannabis has gained a wide reputation of being recalcitrant to gene transformation and tissue culture, a few reports are describing the methods on gene transformation and regeneration (Feeney and Punja, 2003; Slusarkiewicz-Jarzina et al., 2005; Sirkowski, 2012; Wahby et al., 2013; Schachtsiek et al., 2019). Genome editing holds the potential to develop knockout mutants for significant cannabinoid biosynthesis genes such as THCA synthase, CBDA synthase, and CBGA synthase. Several varieties were tested; most were monoecious, although a few dioecious varieties were also used. In all cases, *Agrobacterium*-mediated gene transfer system was employed and exhibited successful transfer of genes, but the regeneration frequency was low to none. Feeney and Punja (2003) demonstrated the transformation success at the cellular level, but none of their treatments were successful in regeneration. Similarly, Wahby et al. (2013) applied *A. rhizogenes* strains (A4, AR10, C58, and IVIA251) and could induce hairy roots on the explants derived from hypocotyl and cotyledonary node; however, plantlet regeneration became a bottleneck for them as well. There is two patent information with the claim of successful genome modification and regeneration of cannabis with limited descriptions (Sirkowski, 2012). Thus, there is a need for an optimized protocol for the transformation and regeneration of cannabis replicable and reliable across different species.

#### Transient Genetic Transformation

There are various molecular tools developed for transient genetic transformation, including virus-induced gene silencing (VIGS). VIGSis an RNA mediated post-transcriptional gene silencing (PTGS) technique applied to study gene function in a relatively short period (Baulcombe, 1999; Liu et al., 2002; Senthil-Kumar and Mysore, 2014; Adhikary et al., 2019). Once a VIGS protocol is established in a species, it takes 3–6 weeks to see the loss-of-function phenotype of the tested gene/s *in vivo* (Adhikary et al., 2019). Thus, this is an ideal tool to apply, as a proof of concept, to define a target gene’s function prior to creating a stable transformation. VIGS, using the Cotton leaf crumple virus (CLCrV), was recently established in *C. sativa*, demonstrating the loss-of-function phenotype of *phytoene desaturase* (*PDS)* and *magnesium chelatase subunit I* (*Chll)* genes (Schachtsiek et al., 2019). Although the loss-of-function phenotype was weak, the researchers paved a clear path to explore unknown genes’ functions in the species. There are viral pathogens reported in cannabis (McPartland, 1996) and many viral vectors developed to date; tobacco rattle virus (TRV) is one of them with a broad-spectrum host range (over 400 plant species) across dicot species (Dinesh-Kumar et al., 2007). Given that TRV can also infect cannabis, potentially demonstrating a strong loss-of-phenotype than CLCrV viral vector.

#### Stable Genetic Transformation

Both transient and stable transformations have been incredibly beneficial for different research areas and applications in functional genomics. Stable gene transformation is preferred for many applications because once the gene modification is fixed in a plant system, it is heritable. The advantage of the altered gene function can be reaped for generations. As there are numerous reports of successful CRISPR-Cas9 mediated gene editing in many plant species, adopting this newly developed molecular tool in cannabis is vital to improving this economically important plant species. CRISPR can precisely alter a gene’s function in a genome (Jinek et al., 2012). It has great potential to benefit both basic and applied plant biology research and development. Therefore, establishing the technology in the cannabis crop is essential for functional studies of thousands of unknown genes and the development of novel varieties.

Traditional genetic modification (GM) and gene editing by CRISPR method are viewed differently (Shew et al., 2018). Gene editing performed using CRISPR method is not considered to be GM organism in some regions. Conventionally, GMO crops refer to organisms that have been altered in a way that they would not have evolved naturally. Moreover, GMO involves transferring foreign DNA fragment from one species to another (transgenic) or within the same species (cisgenic). But in the case of CRISPR edited plants, the targeted mutation is created by using an enzyme and a small guide RNA. While the mutation continues to inherited, the CRISPR machinery can be eliminated in the next generation (Aliaga-Franco et al., 2019). This method is precise and faster than conventional breeding practices, and it is much less controversial than GMO techniques. Therefore, the establishment of CRISPR-Cas9 system in cannabis is another crucial aspect that needs to be explored.

### Hairy Root Culture

*Agrobacterium rhizogenes* is another functional genomics tool to assess the function of a gene or developing transgenic plants. These are differentiated cultures that are induced by the infection of *Agrobacterium rhizogenes*, a soil bacterium. Hairy root culture has a high growth rate in a hormone-free medium and exhibits the potential to yield secondary metabolites comparable to the wildtype (Pistelli et al., 2010). It enables the use of stable and reproducible bioreactor-based production and extraction independent of weather conditions, regulatory hurdles, and a lower risk of microbial contamination. This is a safe approach for producing medicinal and active metabolites free of hormones/viruses and does not require pesticides or insecticides. It is also one of the critical avenues for cannabis genetic transformation and functional genomics research.

Calli or hypocotyls infected by *A. rhizogenes* respond with the emergence of hairy roots from the infected site. Hairy roots can be individually selected and tested for a higher production rate of a compound of interest and cryopreserved at –196°C as a pure culture and subculture further for commercial-scale production (Engelmann, 2004). Cannabis hairy root culture has been successfully induced by *A. rhizogenes* (Wahby et al., 2006, 2013). Hairy root cultures from cannabis callus were also reported using 4 mg/l NAA as a supplement to B5 medium under dark conditions at 25°C (Farag and Kayser, 2015). In the study, the level of THCA and CBDA was less than 2 μg/g dry weight indicating a very low level of cannabinoids present in the hairy root culture under the dark condition with a 28-day growth cycle.

While detectable levels of cannabinoids are not present in *C. sativa* hairy roots, they have been reported to contain choline, atropine, and muscarine (Wahby et al., 2006, 2017). A higher level of these compounds was observed in the *A. rhizogenes* transformed hairy roots compared to non-transformed control. Choline was the most significant compound ranged between 203 and 510 mg/L (control 66–153 mg/L); Atropine with 562–933 μg/L (control 532–553 μg/L); Muscarine with 231–367 μg/L (control undetectable) (Wahby et al., 2017). Additionally, the THCA synthase gene’s heterologous expression in tobacco hairy root culture has been successful (Sirikantaramas et al., 2004; Taura et al., 2009).

### Meristem Culture

The culture of indeterminate organs, especially the totipotent cells in the apical dome, is a method to obtain many virus clones in a short period (Mori, 1971; Wang and Charles, 1991). The apical dome region has no vascular connection to the developing procambium, leaf primordium, and axillary buds (Wang and Charles, 1991). This lack of vascular connection provides a basis for using the meristem for pathogen elimination as viruses readily travel through the vascular system but do not efficiently transfer from cell to cell. Uninfected cells can be isolated from the meristematic dome (Wang and Charles, 1991; Wu et al., 2020). It is a robust tool for producingvirus-free clones that can then be further multiplied at a commercial scale to produce certified virus-free plants. Characteristically, a section of tissue, mostly the apical dome, is dissected either from apical or lateral buds consisting of leaf primordia (no more than 1–2 in number) and apical meristem (0.1–0.5 mm in length) and cultured in a suitable growth medium. Upon induction of the meristem cells under a favorable combination of hormones and growth environment, the cells can continue to develop into a shoot or regenerate into plants through somatic embryogenesis or shoot organogenesis. The regeneration process occasionally gives direct shoot development from the explant, and sometimes morphogenesis occurs indirectly only after the formation of the callus.

There are well-established meristem culture protocols for different model and non-model species (Mori, 1971; Mordhorst et al., 2002; Al-Taleb et al., 2011; Spanò et al., 2018), including the closest relative of cannabis, *Humulus lupulus* (Hops), for eliminating virus infection (Grudzinska and Solarska, 2004; Grudzinska et al., 2006; Adams, 2015; Sallie and Jones, 2015). Given the importance of cannabis as a crop, the development of meristem culture for clean plant production could be useful. Unfortunately, this technique is most effective with viral diseases and would not eliminate fungal and bacterial pathogens known to infect cannabis.

### Protoplast Culture

For decades, plant protoplasts have been used for genetic transformation, cell fusion, somatic mutation, and more recently, for genome editing (Lei et al., 2015). Significant progress has been made in other crop species in genetic studies using protoplasts; however, for cannabis, studies are in a development phase, with the conditions suitable for the survival of transfected protoplasts and plant regeneration are yet to be optimized. Mesophyll protoplast isolation and transformation of at least three different cannabis cultivars has been reported (Morimoto et al., 2007; Flaishman et al., 2019). Based on the recent study, only about 4% of the protoplasts survived 48 h in liquid culture and plants were not regenerated (Flaishman et al., 2019). Even in the absence of successful regeneration of a whole plant, protoplasts are of great value in confirming the effectiveness of designed guide RNA (gRNA) prior to their use for the regeneration of gene-edited plants.

### Somatic Embryogenesis

Somatic embryogenesis is the regeneration of a whole plant from cultured plant cells via embryo formation, from somatic plant cells of various tissues like root, stem, leaf, hypocotyl, cotyledon or petiole (Shen et al., 2018). They morphologically resemble the zygotic embryo’s bipolar structure, bear specific embryonic organs, and go through analogous development stages with similar gene expression profiles (Shen et al., 2018). Somatic embryogenesis can occur through direct regeneration. The embryos are developed directly from explant cells, or more commonly through indirect regeneration in which callus develops first, and the development of embryos occurs from callus cells (Sharp et al., 1980).

Plant regeneration via somatic embryogenesis starts with the initiation of embryogenic cultures by culturing various explants on media supplemented with only auxins or a combination of auxins and cytokinins to control cell growth and development (Osborne and McManus, 2005). One exception to this is the use of thidiazuron (TDZ), a cytokinin-like compound that is often used alone to induce somatic embryogenesis (Murthy et al., 1995). The proliferation of embryogenic cultures can occur on solid or in liquid media supplemented with auxins and cytokinins, followed by pre-maturation of somatic embryos on lower levels of PGRs or PGR free media to stimulate somatic embryo formation and development. Maturation of somatic embryos can occur by culturing on media with reduced osmotic potential or supplemented with abscisic acid (George et al., 2007). This maturation stage is critical for synthetic seed production as it allows embryos to be desiccated, stored, encapsulated, and treated like regular seeds. However, in many somatic embryogenesis systems, the maturation phase has not been developed, and somatic embryos germinate precociously to produce plants.

Somatic embryos are used as a model system in embryology studies; however, somatic embryogenesis’s main economic applications are for developing transgenic plants and large-scale virus-free vegetative propagation of elite plant genotypes. The possibility to scale up the propagation using bioreactors has been reported (Hvoslef-Eide and Preil, 2005). Somatic embryos are also ideal for genetic manipulation purposes as they develop from a single cell, thereby reducing the chances of producing chimeric plants, common when relying on shoot organogenesis or shoot proliferation (Dhekney et al., 2016). Other less common uses of somatic embryogenesis include cryopreservation of genetic materials and synthetic seed technology (George et al., 2007).

Feeney and Punja (2003) investigated the somatic embryogenesis and tissue culture propagation of hemp. Despite testing various explants and supplements, and variations in the culture medium and changes to the culture environment, there was no successful plantlet regeneration, and a reliable protocol for somatic embryogenesis in cannabis has yet to be published.

### Thin Cell Layer (TCL)

Thin cell layer (TCL) culture utilizes a thin layer of tissue as the explant to allow close contact between wounded cells and nutrients and growth regulators supplied in the medium; this controls the morphogenesis of the cultures (Nhut et al., 2003). This is most useful where larger explants may also contain a high level of endogenous hormones, carbon sources, and other substances that influence and conflict with the effects of exogenous substances placed in the medium and, thus, interfere with development. In general, sterilized TCL explants are excised either longitudinally (0.5–1 mm wide, 5–10 mm long) or transversally (0.1–5 mm thick) prior to culturing (Nhut et al., 2003; Croom et al., 2016). Like other *in vitro* techniques, TCL requires an optimized protocol regarding basal media, PGRs and other added nutrients and growth conditions such as daylength, light intensity, and temperature. These conditions vary for not only the species but can be genotype-dependent. It has been widely used in different species, including bamboo, banana, citrus, tomato, rose, *Lilium ledebourii*, *Bacopa monnieri*, saffron, among others (Nhut et al., 2003; Teixeira da Silva et al., 2007; Mirmasoumi et al., 2013; Croom et al., 2016; Azadi et al., 2017). TCL’s potential is yet to be explored in *Cannabis* spp.; however, it may prove to have some utility in the regeneration of genetic transformants in this high value but re calcitrant regeneration crop.

### Doubled Haploid Production

Androgenesis is a biological process by which a whole plant regenerates directly from immature pollen (microspores) through the embryogenesis developmental pathway under *in vitro* conditions. While the resulting plant is haploid and inherently sterile, a diploid plant can arise either spontaneously or artificially (Gilles et al., 2017), usually with colchicine, which blocks cytokinesis without blocking chromosome doubling (Galazkajoa and Niemirowicz-Szczytt, 2013). This doubled haploid is homozygous at all loci. Doubled Haploid (DH) plants have been extensively used in plant breeding programs to increase the speed and efficiency with which homozygous lines can be obtained (Alisher et al., 2007). DH technology is traditionally used to genetically stabilize parental lines for F_1_ hybrid production. This is important for the rapid integration of new traits through backcross conversion and to develop molecular mapping populations. It is also used to fix desired traits obtained through transformation or mutagenesis and simplify genomic sequencing by eliminating heterozygosity (Ferrie and Mollers, 2011). As such, this technology would be an important tool for both forward and reverse functional genomics studies.

There are two different approaches to develop haploid plants. First, *in situ* methods, using particular pollination techniques such as irradiated pollen, inter-species crosses or so-called ‘inducer lines’ (Ren et al., 2017); second, *in vitro* methods including the culture of haploid cells (gametes) and their development to haploid embryos and consequently haploid plants through germination. The microspores, which can be harvested in large numbers (millions), are generally isolated for culture as a uniform population. Alternatively, the culture of whole anthers is used to obtain haploid plants through the androgenesis process. The main disadvantage of another culture is the potential for developing a mix of both haploid and diploid plantlets (Elhiti et al., 2010). In this review, we will focus only on the production of doubled haploids from microspores using *in vitro* culture.

One of the most important factors affecting DH production is the microspore developmental stage. It is a complicated factor that has a strong influence on microspore culture’s success. It has been reported that only microspores that are at a stage sufficiently immature have the ability to change their developmental fate from a gametophytic to embryogenic, leading to sporophytic development (Soriano et al., 2013). The most amenable stage is either the uni-nucleate stage of the microspore or the early binucleate stage, either at or just after the first pollen mitosis. At this developmental stage, the microspore’s transcriptional status may still be proliferative and not yet fully differentiated (Malik et al., 2007). Although all microspores within an anther would be roughly of a similar age, not all cells have embryonic competence. Therefore, the incremental differences in the stages of development of individual microspores can be considered significant. To avoid this problem, Bhowmik et al. (2011) introduced a new treatment, discontinuous Percoll gradient centrifugation, to provide a uniform population of *B. napus* isolated microspores at the appropriate stage of development. This approach has consistently produced high embryo yields and consistent embryo development.

#### Hemp Microspore Culture

In 2019, an extensive hemp breeding program was introduced at Haplotech Inc.^1^. As there has been no previously reported success in the area, a hemp DH project was initiated to accelerate this program. Four different Haplotech genotypes were used for this experiment. Both male racemes and pollen-induced female colas were collected, and the buds were fractionated according to size into three groups (2–3, 3–4, and 4–5 mm). Each group was surface sterilized with 15% commercial bleach and washed three times with distilled-sterilized water for 5 min each. The sterilized buds were macerated in isolation media (MS basal fortified by 13% sucrose). The isolated microspores were washed by extraction medium two times or until the supernatant became clear. The isolated microspores were subjected to fractional centrifugation using Percoll, as described by Bhowmik et al. (2011). The concentration of microspores was diluted to 4 × 10^4^cells/ml with MS basal fortified by 10% sucrose. Five ml of this diluent (4 × 10^4^) microspores were mixed with 5 ml of induction media (MS basal, 10% sucrose supplemented with different additives for induction) in 47 mm Petri dishes. The final concentration of the culture used was 2 × 10^4^ cells/ml. The isolated microspores in culture were observed every 3 days using an inverted microscope and a binocular microscope.

**FIGURE 5:**
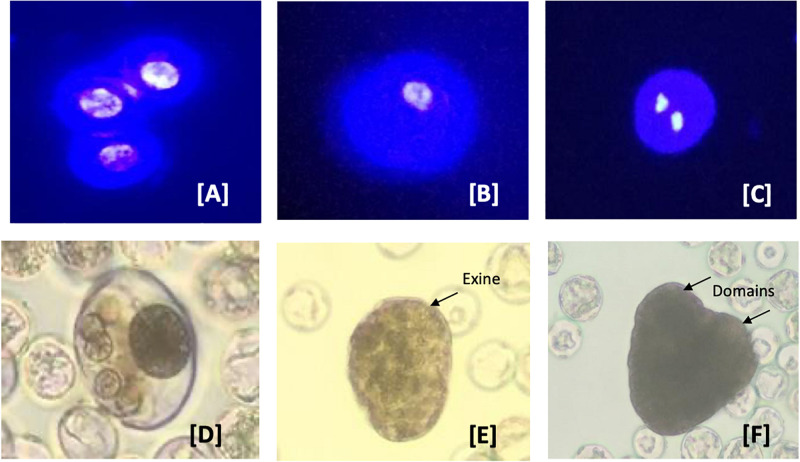
Developmental pathways observed in *C. sativa* (industrial hemp) microspore culture. **(A–C)** Male gametophyte development in *C. sativa* during *in vitro* culture. **(A)** Uninucleate microspores; **(B)** uninucleate microspores after 3 days in culture media; **(C)** symmetrically divided microspore with two equally sized nuclei; **(D)** multinucleate structure without organization and still enclosed in exine; **(E)** globular multicellular structure with developing exine; and **(F)** heart-shape embryo with two distinct domains. The nuclei in **(A–C)** are stained with the nuclear dye 4′,6-diamidino-2-phenylindole (DAPI) to indicate viability.

Samples of isolated microspores were stained with 4, 6-diamidino-2-phenylindole (DAPI) and observed using a fluorescence microscope to monitor their *in vitro* development, once every 3 days. Monitoring of the culture samples by DAPI staining in the first 2 weeks revealed that the microspores of all four genotypes remained uninuclear (Figure 5A). This developmental stage was found to be the most responsive to embryogenesis induction in many crop plants (Soriano et al., 2013). Of the factors tested, the most crucial for further development of the microspore was the induction medium formulation. Using a relatively complex medium, a few microspores responded (0.05–0.5%) and developed further, while the remainder died within 5–10 days. Microspore derived embryos initiated by a series of random divisions within the surrounding exine wall. The nucleus of uninucleate microspores (Figure 5A) condensed and reduced in size during the first 2 days in culture (Figure 5B). They then divided symmetrically within the first 5–8 days, forming two equal-sized nuclei (Figure 5C). This developmental stage is considered the initial stage that is often referred to as sporophytic growth (Soriano et al., 2013). Within another 3–5 days, the nuclei underwent a series of divisions resulting in the formation of multinucleate structures (Figure 5D). By approximately the third week of culture, globular stage embryos were observed in culture (Figure 5E). Early in the fourth week, these globular structures developed into heart stage embryos (Figure 5F). To date, growth has not progressed past this stage of embryo development. Current experiments including adjustment of the osmoticum and removal of secondary metabolites which could inhibit (microspore-derived) embryo development are running.

#### *In vitro* Mutagenesis

A mutation occurs in DNA, naturally or it can also be induced artificially. The majority of the genetic variation existing in a gene pool has occurred naturally. These genetic variations can be recombined through conventional breeding practices to develop a novel variety with desired gene traits. Although these spontaneous mutations are frequent, the desired mutation in the desired gene segment altering its biological role is extremely rare. Therefore, mutation induction tools are used in the rapid development of genetic variability in crops. For the last few decades, there were several scientific reports published assessing the impact of an induced mutation in the improvement of crops (Brock, 1971; Broertjes and Van Harten, 1988; Micke, 1999; Oladosu et al., 2016). However, in cannabis research and development is rapidly flourishing, but there are only a few reports on targeted mutation through genetic transformation (Feeney and Punja, 2003; Slusarkiewicz-Jarzina et al., 2005; Sirkowski, 2012; Wahby et al., 2013) and there is no mutant variety introduced at the commercial level. *In vitro* culture techniques, coupled with mutagenesis, has simplified the crop improvement work for both seeds and vegetatively propagated plants (Hussain et al., 2012). Little efforts have been made and published to establish DH production in cannabis, but once streamlined will open up exciting opportunities for DH mutagenesis as it has been successfully employed in canola (Szarejko, 2003).

### Synthetic Seed Technology

**FIGURE 6:**
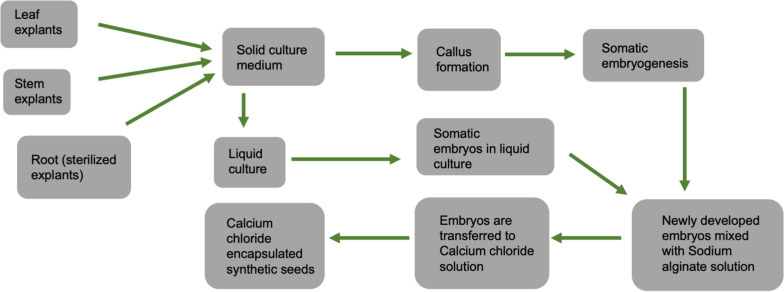
General schematic diagram showing steps for calcium chloride encapsulated synthetic seed production.

Synthetic seeds usually refer to artificially encapsulated somatic embryos (Murashige, 1977) but have also been used in reference to encapsulated vegetative tissues that have the potential to develop into a whole plant (auxiliary buds, cell aggregates, shoot buds). Somatic embryos provide the ideal approach to developing synthetic seeds as they often have the ability to survive desiccation and can be treated in much the same way as true seeds. At the same time, other tissues lack this capacity and are less useful (Rihan et al., 2017). As shown in Figure 6, synthetic seeds can be successfully developed by using various explants, media, and encapsulation protocols (Bapat et al., 1987; Corrie and Tandon, 1993; Nyende et al., 2003; Chand and Singh, 2004; Rai et al., 2008; Lata et al., 2009a).

Cannabis is generally a cross-pollinating crop, and due to its allogamous nature, it is difficult to maintain existing elite varieties by seed. Typically, a minimum isolation distance of 5 km between breeding nurseries and hemp production fields is required to minimize the occurrence of nuisance pollen. Such separation is often difficult to achieve in areas with high hemp production intensity. Therefore, *in vitro* propagation using synthetic seed technology is an alternative method for large-scale clonal propagation and germplasm preservation. As the cannabis industry grows, this method may be cheaper and faster than traditional tissue culture methods. Along with the preservation of genetic uniformity, clones produced through this technique are pathogen-free, easy to handle, and transport.

Moreover, in other species, this approach has resulted in increased quality of planting material (Rihan et al., 2017). While cannabis tissue culture methods are still being optimized, Lata et al. (2009a) developed a high-frequency propagation of axillary buds of *C. sativa* encapsulated in calcium alginate gel. Calcium alginate is a hydrogel that contains nutrients, growth regulators, and sometimes antibiotics.

When directly sown on a substrate, encapsulation aids in the physical protection and establishment and growth of the explant. According to Lata et al. (2009a), gel capsule consisted of 5% sodium alginate with 50 mM CaCl_2_.2H_2_O, and full-strength MS medium supplied with 0.5 μM TDZ, and 0.075% plant preservative mixture (PPM). The optimal regrowth and conversion were achieved in MS medium supplemented with antimicrobial components, PPM (0.075%) and TDZ (0.5 μM) under *in vitro* conditions. Under *in vivo* condition, the optimal conversion and regrowth were exhibited on 1:1 potting mix-fertilome with coco natural growth medium supplied with MS medium containing 3% sucrose, 0.5% PPM. Clones regenerated from the explants were successfully hardened and transferred to the soil (Lata et al., 2009a).

Another hurdle to *in vitro* propagation is transporting requested strains from the tissue culture facility to the growers in a timely manner. These transportation issues become incredibly challenging for maintaining crop schedules because cannabis crops can take more than 2 months to reach hardening stages, then spend 4 weeks in vegetative growth, then 7 or 8 weeks in flower. Greenhouse or indoor growers require a consistent supply demand to receive a high volume of plantlets every week to start over a new grow room at a very tight on-time delivery schedule, which is the most important metric in their operations. An established and cost-effective synthetic seed encapsulation technique would provide an opportunity to eliminate the transportation challenge.

## Cryopreservation

Cryopreservation refers to the storage of diverse living materials at below –130°C (Engelmann, 2004). It serves as an alternative conservation approach to the conventional field and *in vitro* (i.e., slow growth) germplasm conservation and is cost-effective over extended periods with minimal space and routine maintenance requirements (Pence, 2011; Engelmann, 2014; Popova et al., 2015). It also assists current breeding programs by providing long-term storage and an easy long-distance exchange of genetic materials (e.g., pollen and meristematic apices and buds). Cryopreservation has been implemented for various plant species using different methods, the most popular and widely applicable, including controlled freezing, vitrification, encapsulation-dehydration, encapsulation-vitrification, and droplet-vitrification (Sakai and Engelmann, 2007; Popova et al., 2015). These methods follow distinct approaches to dehydrate cryopreserving living materials by converting liquid water to a glassy state to avoid the lethal formation of intracellular ice. The selection of methods and the scales of conservation using this approach are strongly determined by genotypes and tissue materials used, which contain different responses to pre- and post-cryopreservation treatments.

Conventional and *in vitro* conservation of cannabis require considerable amounts of space and routine maintenance, have genetic mutations accumulate in the plants. Conventional conservation may expose plants to virulence pathogens. The plants may eventually become susceptible to diseases. The application of cryopreservation can serve as an essential tool for the conservation of various valuable *C. sativa* genotypes with unique attributes and trading the genotypes nationally and internationally in sterile conditions. The first study on applying cryopreservation techniques in *C. sativa* was reported in 1989 using cell suspension cultures (Jekkel et al., 1989). The suspension cultures were preserved using 10% dimethyl sulfoxide (DMSO) cryoprotectant and a controlled cooling rate of 2°C/min and transfer temperature of –10°C, with a 58% survival rate after cryopreservation of the cultures. A cryopreservation protocol for *C. sativa* shoot tips was recently developed using a droplet-vitrification in liquid nitrogen for long-term conservation of this crop (Uchendu et al., 2019). The report showed that vitrified shoot tips using a cryoprotectant solution of 30% glycerol, 15% ethylene glycol, 15% DMSO in liquid MS medium with 0.4 M sucrose, pH 5.8 had 63% re-growth efficiency. Despite the promising progress made, more studies need to be done on selecting appropriate cryopreservation methods with respect to the tissue types and genotypes, increasing re-growth and survival efficiency of preserved samples, and genetic stability of regenerated plants after using different cryopreservation tools, among others.

## Germplasm Maintenance

The *in vitro* condition also raises some issues for concern, primarily when the material is maintained over a long period of time.

### Clonal Stability *in vitro* Culture

*In vitro* mass-propagation and maintenance of elite germplasm requires genetically stable true-to-type clones. Several factors, such as the number of subcultures, changes in the relationship of auxin/cytokinin, explant type, and a high concentration of growth regulators, may influence the genetic stability of a clone under *in vitro* conditions (Joyce et al., 2003; Sato et al., 2011; Smulders and de Klerk, 2011; Nwauzoma and Jaja, 2013). While carefully selecting explant types and optimizing the conditions above, but depending on the plant species, clonal stability can be obtained during *in vitro* mass-propagation and germplasm conservation of the desired elite genotypes maintained. To date, *C. sativa* plants regenerated from nodal culture, and *in vitro* conserved synthetic seeds (‘Encapsulated’ nodal segments) have shown no evidence of genetic mutations; however, this has only been evaluated using low numbers of markers (Lata et al., 2010a, 2011). Despite optimizing and using properly *in vitro* conditions that limit somaclonal variations, assessment of clonal stability is required to ensure the regenerated clones are the true-to-type of the donor plants.

### Somaclonal Variation

Although clonal propagation and maintenance of elite germplasm require a substantial genetic uniformity among *in vitro* regenerated plantlets, there may be a large possibility of genetic variations, called “somaclonal variation” among these plants and/or relative to the donor plants. Somaclonal variation is commonly a result of genetic alterations and changes in the new *in vitro* plants’ epigenetics compared to the original source plants (Miguel and Marum, 2011; Abreu et al., 2014). The frequency and nature of somaclonal variation *in vitro* culture can be influenced by different factors, such as explant source, genotype, *in vitro* techniques, *in vitro* growth conditions, length of the culture period, and the number of subcultures. The use of *de novo* regeneration from highly differentiated tissues (i.e., roots, leaves, stems, hypocotyls, cotyledons, etc.) is generally considered to produce more somaclonal variation compared to explants with developed meristems (i.e., axillary buds and shoot tips) (Pijut et al., 2012). Most of these factors generate oxidative stress during culture initiation and subsequent subculturing. The explants and the subsequent regenerated plants exposed to the stress may retain genetic changes. For example, protoplast and callus based plant regeneration impose a high degree of oxidative stress; thus, the stress promotes a high mutation rate, whereas plants regenerated through auxiliary branching (e.g., nodes, shoot tip) experience very low oxidative stress, normally resulting in no genetic variation (Zayova et al., 2010; Smulders and de Klerk, 2011; Krishna et al., 2016). Genetic variation can also arise from somatic mutations already present in the explants collected from the donor plant (Karp, 1994). *In vitro* regeneration of plants can also be genotype-specific, in which genotypes have different degrees of mutation risks and thus strongly determine the formation of somaclonal variation (Alizadeh et al., 2010; Eftekhari et al., 2012; Nwauzoma and Jaja, 2013). The genetic alterations strongly depend on the *in vitro* techniques used to regenerate *in vitro* plants. Additionally, despite differences across plant species, cultures maintained for a long period tend to generate high somaclonal variation, and *vice versa* (Farahani et al., 2011; Jevremovic et al., 2012; Sun et al., 2013). When cultures are getting old and continuously subcultured, the chance of generating genetically less uniform plants is increased (Zayova et al., 2010), but depends upon the plant species. For example, any more than eight subculture cycles increased somaclonal variation in banana (Khan et al., 2011), whereas over 30 subcultures did not cause any detectable somaclonal variations in *C. sativa* (Lata et al., 2010a).

Although the molecular mechanism of how somaclonal variations generated from a single plant genotype under the same *in vitro* conditions is not fully explored, several potential mechanisms causing genetic alternations and epigenetics have been proposed in different plant species. These mechanisms include changes in chromosome number, point mutations, somatic crossing over and sister chromatid exchange, chromosome breakage and rearrangement, somatic gene rearrangement, DNA replication, changes in organelle DNA, insertion or excision of transposable elements, segregation of pre-existing chimeral tissues, DNA methylation, epigenetic variation, and histone modifications and RNA interference (Sato et al., 2011; Krishna et al., 2016; references therein).

The occurrence of somaclonal variations in regenerated *in vitro* plants may be advantageous or disadvantageous, depending on *in vitro* propagation goals. If *in vitro* propagation aims to generate new variants, obtaining variations among *in vitro* plants can be advantageous that increases genetic diversity for a genotype used. It provides an alternative tool to the breeders for obtaining genetic variability in different plant species, which are either difficult to breed or have narrow genetic bases. On the flip side, when *in vitro* propagation targets to produce multiple true-to-type *in vitro* plants and maintain elite germplasm, the occurrence of subtle somaclonal variations is a severe problem.

## Phytocannabinoid Synthesis in the Cannabis Species

Nature has deftly adorned cannabis species with a spectrum of phytocannabinoids or monoterpenoids that are chemically designed with para-oriented isoprenyl and aralkyl groups (Hanus et al., 2016). Since the discovery of tetrahydrocannabinol (THC) and cannabidiol (CBD) in the early 1960s, there are over 120 cannabinoids that has been reported, and the biosynthesis pathway of these compounds has been greatly improved (Taura et al., 1995; Sirikantaramas et al., 2004; Taura et al., 2007b, 2009; Gagne et al., 2012; Stout et al., 2012; Laverty et al., 2019). Presumably, cannabigerolic acid (CBGA), the product formed by the alkylation of geranyl diphosphate and olivetol, is the key precursor compound in the synthesis of cannabinoids (Fellermeier and Zenk, 1998). The cyclization event of prenyl components of CBGA, catalyzed by three enzymes – tetrahydrocannabinolic acid synthase (THCAS) (genebank accession: AB057805), cannabidiolic acid synthase (CBDAS) (genebank accession: AB292682), and cannabichromenic acid synthase (CBCAS), lead to the formation of three major cannabinoids, THCA, CBDA, and CBCA, respectively (Sirikantaramas et al., 2004; Taura et al., 2007a). Biochemical characterization of the enzymes, THCAS and CBDAS, have demonstrated that the enzymes follow a similar reaction mechanism. In the presence of molecular oxygen, the enzymes use flavin adenine dinucleotide (FAD) cofactor to catalyze CBGA forming THCA and CBDA, and hydrogen peroxide as its chemical biproduct (Sirikantaramas et al., 2004; Taura et al., 2007b). Although it is a bit unclear, the chemical reaction for CBCAS also believed to use FAD as cofactor and molecular oxygen to complete the enzymatic activity on CBGA. The genes that encode for CBCAS and THCAS are highly similar in the nucleotide level, indicating that CBCAS is also flavoproteins, like the other two enzymes, requiring oxygen to catalyze CBGA to CBCA (Laverty et al., 2019). THCA, CBDA, and CBCA are the major cannabinoids in acidic forms that are synthesized in cannabis plant; upon decarboxylation, these compounds convert into neutral forms, THC, CBD, and CBC respectively (Wang et al., 2016).

## Determination of Genetic Fidelity

**FIGURE 7:**
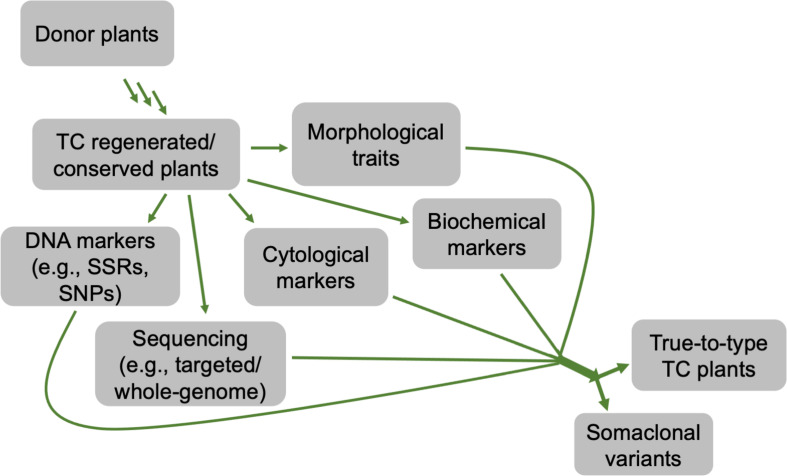
A flow chart depicting different approaches that can be used to determine the genetic stability of *in vitro* regenerated or conserved cannabis plants, compared to its donor counterparts.

Variations between regenerated and donor plants can be exhibited at phenotypic, cytological, biochemical, and genetic/epigenetic levels (Hillig, 2005; Miguel and Marum, 2011; Smulders and de Klerk, 2011; Abreu et al., 2014). These variations can be determined through different approaches, such as morphological, cytological, biochemical, and molecular analyses (Figure 7). For morphological traits, changes are not always observed at early developmental stages or may not entirely display the variations. By contrast, the use of cytological and molecular detection approaches determines differences at chromosomal and DNA levels, respectively, regardless of the developmental stages in various plant species (Clarindo et al., 2012; Pathak and Dhawan, 2012; Currais et al., 2013; Abreu et al., 2014; Bello-Bello et al., 2014). To date, several studies have been reported on the use of different molecular markers in *Cannabis* spp. genetic diversity, fingerprinting, etc. These markers include random amplified polymorphic DNA (RAPD), restriction fragment length polymorphisms (RFLP), amplified fragment length polymorphism (AFLP), microsatellites, inter simple sequence repeat (ISSR), short tandem repeat (STR) multiplex, and single nucleotide polymorphisms (SNPs) and PCR Allele Competitive Extension (PACE) assay (Faeti et al., 1996; Kojoma et al., 2002; Alghanim and Almirall, 2003; Gilmore and Peakall, 2003; Hakki et al., 2003; Datwyler and Weiblen, 2006; Mendoza et al., 2009; Lata et al., 2010a; Gao et al., 2014; Dufresnes et al., 2017; Henry et al., 2018). These molecular markers coupled with cytological and morphological analyses (Abreu et al., 2014) are valuable techniques to ensure the genetic stability of *in vitro* regenerated plants or *in vitro* conserved germplasm of *C. sativa*. To date, only ISSR markers have been used to confirm the genetic stability of *C. sativa* synthetic seeds during *in vitro* multiplication and storage for 6 months under different growth conditions, and *in vitro* propagated plants over 30 nodal subcultures in culture and hardening in soil for 8 months, compared to the corresponding donor plants (Lata et al., 2010a, 2011).

## Projected Contribution of Tissue Culture in the Global Cannabis Industry

The present global cannabis market is worth $340 B^2^. To supply cannabis (medical and recreational) to global consumers, a stable supply chain of quality production and value-added product development still needs to be established. Considering the average annual weighted usage base of 110 g per customer (Canaccord Genuity), the global cannabis demand currently could be around 19-20 M kg per year. Major cannabis consumers are in Europe, North America, South America, Asia, and Oceanic parts of the world, with an estimate of 263 million people using the drug in the previous year (European Consumer Stables Report, 2018; World Drug Report, 2019). To produce 20 M kg of cannabis every year, considering a 40-gm yield per plant, would require 500 M clones/seeds a year. An average price of $10, as, then, the overall present global expected market size for tissue culture clones/manual clones could be predicted around $5B. With intensive indoor cultivation, tissue culture clonal planting material can also reduce the risk of fungal and viral diseases, substantially reducing production cost to under $0.5 per gram to maintain a profitable cannabis production (Table 4). Considering these global demand scenarios, the supply of clean cannabis clones (pest free, and true to type tested) is an important supply chain component essential for the success and future growth of cannabis industry. To sustain and support the industry growth and make the production cost-effective, optimization in the cannabis tissue culture technology is vital.

**TABLE 4:** Comparison between tissue culture cloning and manual cloning in cannabis.

**Parameter**	**Manual Cloning**	**Tissue culture cloning**
Space to produce 1000 cuttings (square meters)	3–5	0.36
Clones processed per person per day (count)	200–250	1500–2000
Multiplication Ratio per month	1–2	4–5
Cost of Production ($)	$3–4	$0.5–1
Clone multiplication in a 3-month cycle	50–80	200–250
Cleanliness	Chances of contamination	Disease, pest, and virus free
Vigor	Chances of reduced vigor from stressed or infected mother plants	Vigor from meristematic reviving
Estimated clone production per 10,000 square feet per year (count)	200,000	2,000,000
Estimated revenue at $10 per clone	$ 2M	$ 20 M

The *in vitro* propagation of cannabis is superior to conventional methods because of disease-free elite plants’ production and a high multiplication rate. The cannabis industry is keen to invest in *in vitro* propagation due to (i) saving footprint/production area by shifting a mother room to a tissue culture lab that will be almost 10% the size of the space needed same number of clones.

The main hurdle of *in vitro* propagation is the capital cost for the tissue culture lab setup. Setting up a massive large-scale production facility can involve a multimillion-dollar investment. Industry and technology will need to continue to improve and reduce costs so that *in vitro* propagation can be affordable for all growers.

In other plants, under a laminar flow hood setting, on an average of 100 plants per hour with 2000 working hours, 200,000 plants can be produced in a year. With an hourly labor cost of $35 per hour will cost around $0.35 per tissue culture plant (Sluis, 2005). This is around 60% of the production cost, adding another $0.15 for other costs (including electricity, resources, and marketing) makes it a baseline cost of $0.50 per plant. Scale also makes some impact on the cost of production being larger facilities can reduce the cost per plant significantly. These production costs can be as low as $0.15 per plant if the plants are produced in India, Singapore, China, or Africa where labor costs are comparatively low.

**FIGURE 8:**
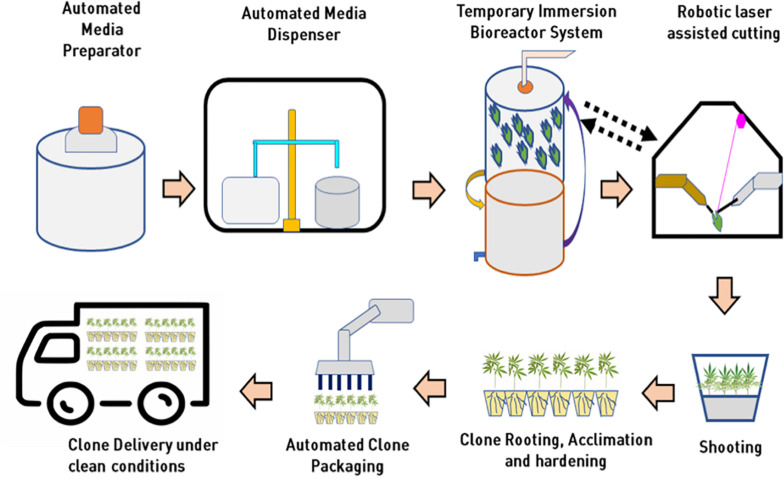
Integration of automation and bioreactor technologies for mass propagation in cannabis for low cost clonal multiplication at *in vitro* level.

A few biotech companies recently added robotic sub-culturing technology for their cannabis plantlets and developed a fully automated micropropagation system to reduce large-scale operation costs. However, the capital investment to purchase this kind of robotic system is incredibly high at this time. Automated technologies for media preparation and dispensing, photoautotrophic bioreactor systems, robotic explant handling, and cutting, transfer laser dissected explants into fresh culture media, and automated acclimatized and hardened plant packaging in future will make cannabis tissue culture industry high throughput and extremely cost-effective for assured “*Just In Time*” supply of pest free, true-to-type cannabis clones. A conceptual model for high throughput automated cannabis *in vitro* clonal mass propagation is depicted in Figure 8. Robotics has the potential to bring tissue culture cost down by 25% (as low as $0.15 per plant to compete with low-cost production in some parts of the world). Tissue culture automation technology is slowly progressing, and it will not only bring high-level consistent output but also reduce the cost of production as low as 20 cents per plant.

## Conclusion

The process of developing new varieties through conventional breeding can take 7–12 years, depending on crop species. The progress of cannabis breeding programs is limited due to the difficulty in maintaining selected high yielding cross-pollinated elite genotypes under field or greenhouse conditions. Therefore, tissue culture techniques are advantageous for cannabis improvement because they can facilitate high multiplication rate and production of disease-free elite plants by overcoming the problems of heterozygosity from cross-pollination. The development of new industrial hemp and medical cannabis cultivars with improved traits could be further advanced using genome editing and other precision breeding tools, combined with *in vitro* techniques for regeneration. Unfortunately, hemp and cannabis plants’ dioecious nature complicates the efforts toward the improvement of specific traits, such as resistance to pests and diseases. Therefore, with the recent legalization, calls for serious targeted efforts are required to advance the regeneration and transformation protocols aiming to enhance the quality and safety of the plants and end products.

## Author Contributions

All authors listed have made a substantial, direct and intellectual contribution to the work, and approved it for publication.

## Conflict of Interest

ME and RG were employed by the company Haplotech Inc. The remaining authors declare that the research was conducted in the absence of any commercial or financial relationships that could be construed as a potential conflict of interest.

## Footnotes

1 https://haplotech.com/

2 https://www.gbnews.ch/340-billion-the-global-cannabis-market/
